# Neoplastic plasma cells with Auer rod‐like inclusions

**DOI:** 10.1002/jha2.33

**Published:** 2020-07-01

**Authors:** Nordberg Nørgaard Jakob, Bugge Askeland Frida, Tjønnfjord Geir, Schjesvold Fredrik

**Affiliations:** ^1^ Department of Haematology Oslo University Hospital Oslo Norway; ^2^ Institute of Clinical Medicine University of Oslo Oslo Norway

A 52‐year‐old man was diagnosed with smouldering myeloma. Two years later, magnetic resonance imaging revealed numerous focal lesions in the spine, pelvis and femora. He was slightly pancytopenic with normal renal function and normal plasma calcium. Protein electrophoresis showed monoclonal IgG kappa of 11 g/L, free light chain kappa of 197 mg/L and free light chain lambda of 9.7 mg/L. Fluorescence in situ hybridization (FISH) analysis revealed the translocation t(11;14)(q13;q32).

**FIGURE 1:**
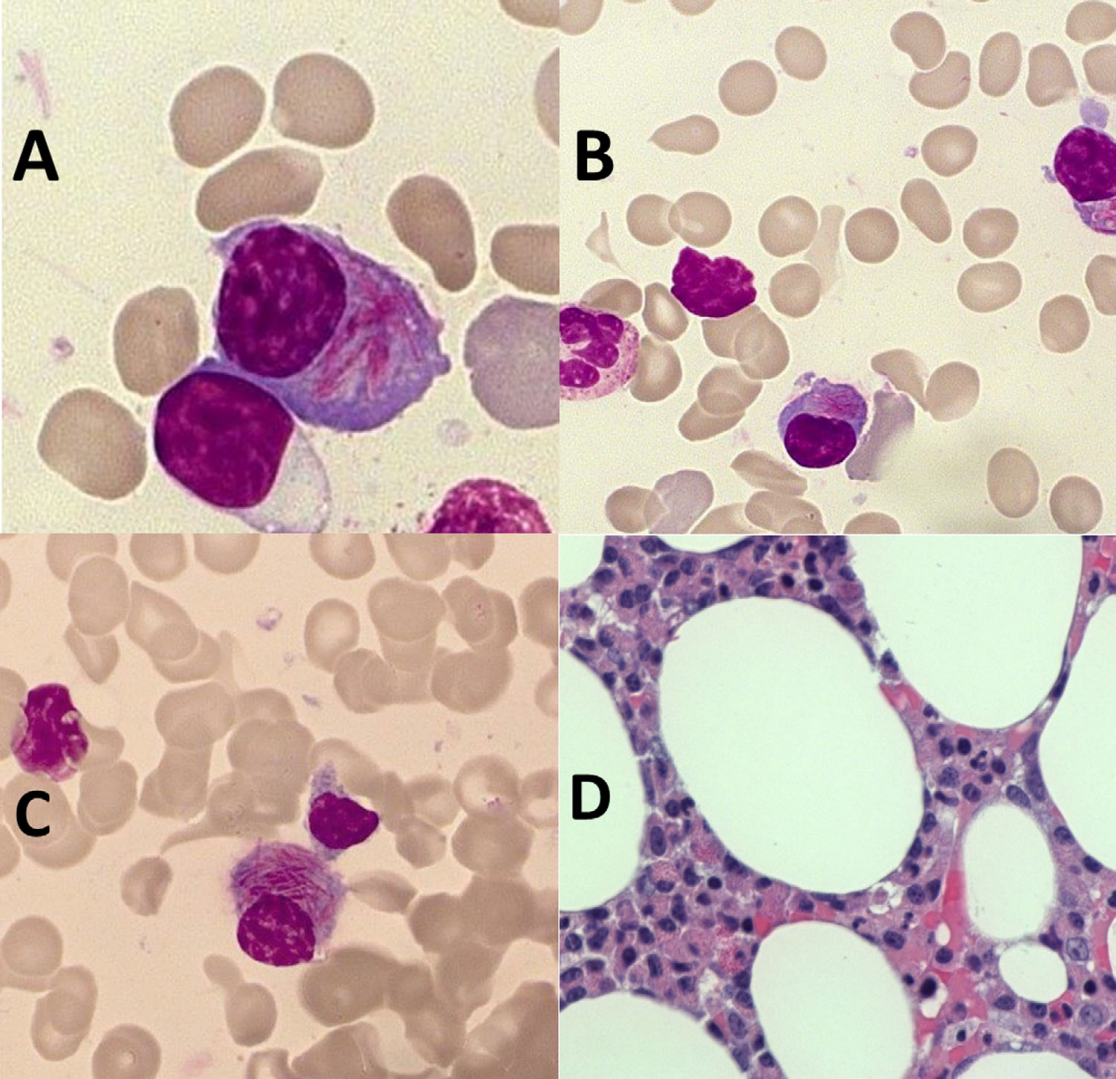
Plasma cells with Auer rod‐like inclusions in bone marrow aspirate (A‐C) and biopsy (D)

A bone marrow smear and biopsy displayed 30‐40% plasma cells. Strikingly, most of the plasma cells had numerous azurophilic needle‐shaped cytoplasmic inclusions (Figure 1). These inclusions resembled Auer rods in a quantity which is only observed in acute promyelocytic leukaemia.

Auer rod‐like inclusions in the cytoplasm of plasma cells are rare. In all described cases, including ours, the monoclonal component is kappa restricted [1]. The nature of these inclusions is not fully unravelled. Cytochemical staining has revealed high alpha‐naphthyl esterase activity in the inclusions indicating that the inclusions may originate from lysozymes [2]. It is unknown whether the inclusions are of any prognostic significance, but an association with Fanconi syndrome has been reported. No sign of Fanconi syndrome was found in our patient.
